# Capital Punishment

**Published:** 1896-06-20

**Authors:** 


### Modern Sociology.

### CAPITAL PUNISHMENT.

Four people were last week hanged at Newgate, and although no one need have any doubt as to their guilt one cannot but ask the question, For what good purpose was this thing done? Was it merely to punish the criminals, or was it done with the intention of deterring others from such crimes? And was it the best method for either of these purposes?

No doubt in times gone by, when death at the hands of the executioner was but the end of a series of tortures which might well appal even the most hardened, when men were boiled in oil, or burned at the stake, or impaled and left to die slowly of dumb agony, the sentence of death was a real terror to evil-doers. But hanging as now performed is an easy death, a momentary and painless transit out of life, far easier than is the lot of most honest men, and one which many suicides would hail with pleasure. The fact is that a sentence of death depends largely for its punitive effect on the faith which the culprit has in future states and in future punishments, and thus falls with ever-varying effect upon its victims. It fails then to satisfy justice in that it fails to deal equally between man and man or to apportion the punishment to the crime.

Does it then succeed as a deterrent?

Universal experience shows that for a punishment to act as a deterrent it must, above all things, be certain. It is here that the death penalty so egregiously fails. Of all forms of punishment it is the most uncertain. The fact that murder is punishable only by death not only vastly increases the difficulty of obtaining a conviction, but leads so often to a reprieve that the convict remains hopeful to the last, and the public are always left in doubt whether execution will really follow sentence. It is inherent in hanging that it is irrevocable, and this, rather than the severity of the punishment, is what makes juries hesitate in convicting; for although every one may agree that the culprit is worthy of the heaviest penalty if he committed the crime, that "if" is a stumbling block over which justice too often trips, and by means of which many a murderer escapes scot free.

Nor can one wonder at juries allowing even the slightest doubt to influence their verdict when they feel that even the slightest error might lead to a judicial murder; for, notwithstanding all their care, it is undoubtedly the fact that, even in comparatively recent times, many people have been wrongly convicted, and some even wrongly executed. The Home Secretary, speaking in Parliament in 1869, said that within half a-year, out of eleven cases of capital sentences, five had had to be set aside by him. One of the convicts "beyond all question . . . was an innocent man," another was certainly insane, and in three other cases the persons concluded to have been murdered had died from other causes. But Mr. Tallack, in his book on "Penology," throws doubt on the justness of the sentence in the case of one of the six, here referred to, who were hanged. This poor woman was charged with poisoning her husband with arsenic, and the circumstantial evidence was strong against her. She protested her innocence in court, but was not believed. On the morning of her execution she exclaimed, "You are not going to hang me, are you? God knows I am innocent!" and she died protesting her innocence to the last. Some years afterwards, a man in the village where this Mrs. Biggadyke had lived became ill, and, on being informed that recovery was hopeless, he manifested great anxiety, and said, "I cannot die until I make a full confession of my guilt. It was I who poisoned poor Biggadyke. His wife, who was hanged for it, knew nothing of it. I went into the house while she was mixing a cake, and put arsenic into the bowl when she was not looking." So, after all, this poor woman was unjustly sacrificed.

The more fully it is understood that, notwithstanding all their care, innocent people are sometimes hanged, the more reluctant are juries to give a verdict which involves an irrevocable punishment. There is a degree of certainty with which men are satisfied in the ordinary affairs of daily life, and with this degree of certainty juries are properly enough content in coming to a verdict in regard to such crimes as are punished by fine and imprisonment. But when it comes to a matter of taking life juries, rightly or wrongly, ask for something more nearly approaching to that absolute certainty which can only very rarely be attainable, and in default of such certainty the human nature within them gives the prisoner "the benefit of the doubt." Thus it happens that, in many a case a doubt which would not be entertained in reference to a theft will set a murderer free. Statistics on this matter are easy to get, and are sufficiently startling.

During the twelve years from 1881 to 1892 there were in England and Wales 2,105 verdicts of "Wilful murder" returned by coroners' juries. These were followed by 775 trials, which resulted in 322 convictions, but in only 181 executions. So that, as is stated by Mr. Tallack, "only a quarter of those put on trial for wilful murder were hanged, and out of those sentenced to death nearly half escaped that penalty." The chances of escape for female murderers are still more extreme, for during the same years five out of every six convicted female murderers escaped the gallows. Thus any deterrent influence which the death sentence may have is counteracted by its unavoidable and special uncertainty of enforcement.

We cannot close, however, without stating again that death is not the punishment which lawless men most dread, and pointing out how large is the number of those who, so far from fearing death, actually seek it, there being about six times as many cases of suicide as of all other forms of homicide put together. Death is said to be the heaviest penalty known to the law; but death is a mystery almost inconceivable to lawless and ignorant members of the criminal classes. Work and hard labour, however, are things which they know about and hate; and it may be questioned whether a sentence of penal servitude for life would not to many of them seem far more dreadful than a hanging. There is no doubt that murders are rarer than they used to be, just as crime in general is less common. It would be a bold man, however, who would maintain that this was due to the retention of capital punishment.

